# Ten-Year Trends in Otolaryngological Surgery Volumes and the Impact of Infection Prevention During the COVID-19 Pandemic—A National Study

**DOI:** 10.3390/jcm13237190

**Published:** 2024-11-27

**Authors:** Ville-Emil Valajärvi, Satu Lamminmäki, Marie Lundberg, Lena Hafrén

**Affiliations:** 1 Faculty of Medicine, University of Helsinki, 00014 Helsinki, Finland; 2 Department of Otorhinolaryngology–Head and Neck Surgery, Helsinki University Hospital, University of Helsinki, 00029 Helsinki, Finland; satu.lamminmaki@hus.fi (S.L.); marie.lundberg@hus.fi (M.L.); lena.hafren@hus.fi (L.H.)

**Keywords:** otolaryngology, ENT, COVID-19, surgery rate

## Abstract

**Background/Objectives:** This register study elucidates the national trends in the otolaryngologic surgery volume in Finland over a ten-year period. In particular, we investigated whether the pandemic, which had a marked effect on communicable diseases, had an impact on infection-related ear, nose, and throat (ENT) procedures. For reference, we used noninfectious ENT procedures. **Methods:** The data of this study consisted of the volumes of different otolaryngological surgical procedures in Finland from 2012 to 2022. A linear regression model was applied to calculate long-term trends in surgery volumes. The annual predicted and observed surgical volumes of each procedure were compared. In addition, different procedures were compared based on whether they were mostly infection-related, non-infection-related, or both. **Results:** The results revealed that the pandemic altered the trends of many ENT procedures, and during the pandemic, infection-related surgeries declined more than non-infection-related surgeries did. The decline in infection-related surgery volumes seems to have lasted longer than the coronavirus disease 2019 (COVID-19) pandemic itself, as only tympanostomies and mastoidectomies of all the infection-related procedures included in this study have returned to the pre-pandemic trend. Some non-infection-related procedures and procedures with mixed indications also declined during the pandemic and sustained their reduction even in 2022. **Conclusions:** This study provides a nationwide insight into ENT surgery volumes in Finland over a ten-year period. Although causative reasoning cannot be conducted based on this study, it still provides a good indication of how the absence of respiratory viruses and multifactorial societal restriction measures could have a long-lasting effect on the epidemiology and management of many ENT diseases.

## 1. Introduction

The coronavirus disease 2019 (COVID-19) pandemic has had wide-ranging and far-reaching effects on health care systems worldwide. One of the most affected medical specialties has been otolaryngology. In the US at the onset of the pandemic, otolaryngological surgery volumes decreased by more than 80% in outpatient settings and by more than 40% in inpatient settings [1]. In many clinics, postponing elective surgery during the early pandemic caused prompt reductions in ear, nose, and throat (ENT) surgery volumes [1,2,3]. However, later studies suggested that some of the reduction may not have been related to surgery cancelation policies themselves but rather to other restrictive measures, particularly the implementation of nonpharmaceutical interventions (NPIs), such as social distancing, mask wearing, and improved hygiene practices [4,5,6,7]. Especially in ENT, a significant proportion of surgical procedures are related to respiratory infections.

In the US, influenza and human metapneumovirus (hMPV) viruses circulated at historically low levels in 2020 and 2021 [8]. Respiratory syncytial virus (RSV) circulation was also historically low in 2020 but increased atypically during the summer of 2021. The incidence of parainfluenza viruses (PIVs) decreased early in the pandemic and returned to normal in spring 2021 [8]. Non-enveloped viruses such as rhinoviruses (RVs) and respiratory adenoviruses initially decreased in number during the early pandemic but quickly returned to their normal seasonal levels [8,9]. In Canada, the detection rates of influenza A and B, RSVs, PIVs, hMPVs, and adenoviruses decreased substantially amid the early pandemic, whereas enteroviruses and rhinoviruses remained in circulation [10].

In Finland, where this study was conducted, the implementation of pandemic NPIs reduced the most common respiratory viral infections. National lockdown led to shorter influenza and RSV seasons and reduced the number of emergency room visits caused by respiratory virus infections in children [11,12]. The RSV and influenza A and B seasonality also diminished among children from 2020 to 2021, when travel restrictions and mask mandates for adults were in place [13]. When these preventive measures were lifted in September 2021, both influenza and RSV cases returned. However, the peak of the RSV epidemic occurred atypically early [13]. On the other hand, NPIs caused only minimal changes in the spread of rhinovirus in Finland [14].

In France, the total number of ENT cancer surgeries decreased between 2010 and 2021 and more rapidly during the COVID-19 pandemic [15]. However, detailed data on the surgical rates of different ENT procedures are lacking. Here, we aim to assess the long-term trends in separate ENT procedures and evaluate the effects of the altered epidemiology of seasonal viruses during the COVID-19 pandemic.

## 2. Materials and Methods

The data of this study consisted of the volumes of different otolaryngological surgical procedures in Finland from 2012 to 2022. The data were collected from an open-access register of the Finnish Institute for Health and Welfare (THL). In this register, all the procedures performed in Finland are classified according to the Nordic Classification of Surgical Procedures (NCSP) codes. The register contains the annual volumes of 57 different otolaryngological procedures, of which 27 large-volume procedures were included in this study. Based on the judgments of three ENT specialists, the procedures were divided into three categories: infection-related, non-infection-related, or uncategorizable (Table 1). Procedures related to the treatment of otitis media and sinusitis, such as myringotomies, tympanostomy tubes, and sinus lavages, were classified as infection-related, whereas procedures related to the treatment of malignancies, such as laryngectomies and the insertion of cochlear implants, were categorized as noninfectious. Tonsillectomies were classified as infection-related since they are performed mainly because of chronic, recurrent, or complications of tonsillitis, whereas the main indication for a partial tonsillectomy is airway obstruction, and hence it was classified as noninfectious. Some procedures, such as adenotonsillectomies, adenoidectomies, tracheostomies, and middle meatal antrostomies (MMAs), are performed both for infectious and noninfectious diseases and were thus excluded from this binary grouping (Table 1).

Information about the Finnish government’s policy responses to the COVID-19 pandemic was obtained from the Finnish Institute for Health and Welfare and the Ministry of Social Affairs and Health.

Statistical analysis was performed via SPSS (version 28.0; IBM, Armonk, NY, USA). A linear regression model was applied to calculate trends in surgery volumes between 2012 and 2019. This trend was extrapolated to the pandemic years 2020–2022 via 95% prediction intervals. The annual predicted and observed surgical volumes of each procedure were compared, and the percentage change between these two procedures was calculated. The observed values outside of the prediction interval indicated a significant change. To compare infection-related and non-infection-related procedures, a residual for each procedure was calculated by subtracting the predicted value from the observed value. The residuals of procedures in the infection-related and non-infection-related groups were summed. The nonparametric Mann–Whitney U test was used to test whether the summed residuals differed significantly between the groups in 2020, 2021, and 2022. Further, a 2 × 2 contingency table was formed of infection-related and non-infection-related procedures for each year, observed surgery numbers, and the residuals. A Chi-Squared test was used to study whether there was a difference between infection-related and non-infection-related groups.

**Figure 1:**
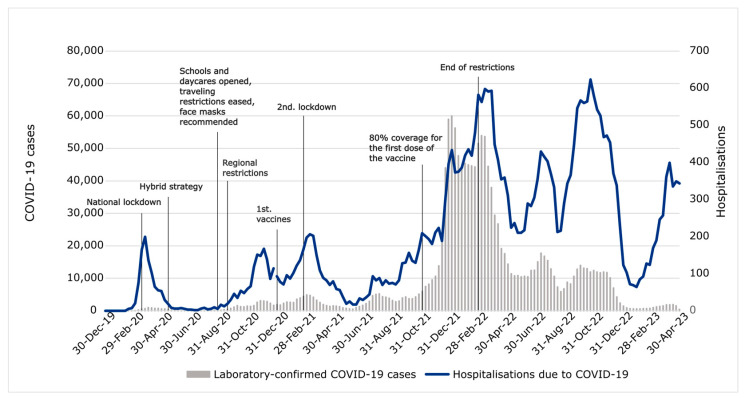
Social restriction measures in Finland during the pandemic, laboratory-confirmed positive COVID-19 tests, and hospitalizations due to COVID-19.

Figure 1 illustrates the course of the COVID-19 pandemic in Finland. Five major hospitalization waves can be distinguished, which somewhat aligns with laboratory-confirmed positive tests. At the onset of the pandemic, social restriction measures were announced at the national level, and restrictions were subsequently made more regionally. Regional restrictions of varying severity were lifted by February 2022. Appendix A contains a more detailed description of the restrictions and vaccinations in Finland during the pandemic [16,17,18,19,20,21].

## 3. Results

Altogether, 384,823 ENT procedures of the selected categories were performed in Finland between 2012 and 2022. Table 1 shows the annual volumes of the 27 types of procedures. Tympanostomies were the most common infection-related surgeries (*n* = 103,413) during the decade, followed by maxillary lavages (*n* = 58,357) and tonsillectomies (*n* = 55,141). Among noninfectious cases, the most common were cervical lymph node biopsies or neck dissections (*n* = 13,569) and partial tonsillectomies (*n* = 11,092).

### 3.1. Infection-Related Procedure Volumes

**Figure 2:**
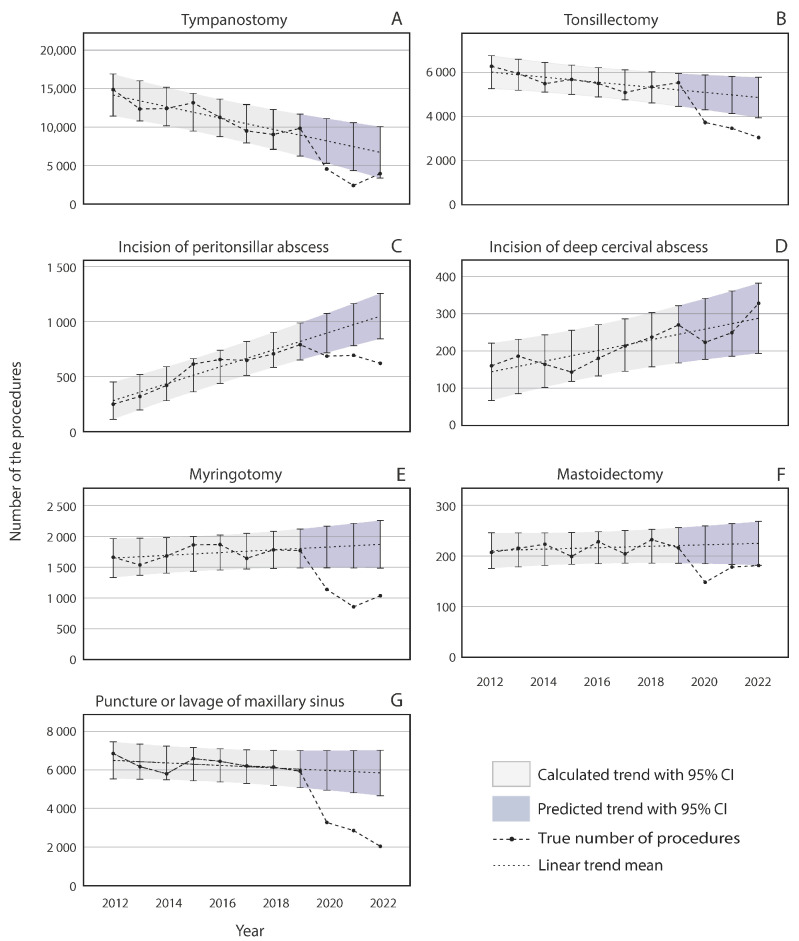
The annual number of infection-related otolaryngologic procedures performed in Finland from 2012 to 2022. The black dots depict the true number of procedures, and the gray and blue areas represent the 95% prediction intervals.

The trends in the volume of infection-related procedures in Finland between 2012 and 2022 are illustrated in Figure 2. Before the COVID-19 pandemic, the annual surgical volumes of tympanostomies (Figure 2A) and tonsillectomies (Figure 2B) declined, whereas the volumes of peritonsillar abscess incisions (Figure 2C) and incisions of deep cervical abscesses (Figure 2D) demonstrated increasing trends. The annual volumes of myringotomies, mastoidectomies, and punctures/lavages of maxillary sinuses remained rather stable between 2012 and 2019 (Figure 2E–G).

Compared with the predicted trend, the pandemic affected all other infection-related procedure volumes negatively except incisions of deep cervical abscesses (Figure 2D), which remained statistically significant for the predicted volumes. Compared with the predicted pre-pandemic trend, the tympanostomy volume decreased by 44.5% in 2020 and further decreased by 67.6% in 2021. However, in 2022, the volumes had recovered to the prediction (Figure 2A). For mastoidectomies (Figure 2F), recovery occurred sooner, with a reduction of 33.3% in 2020 and a smaller but still significant reduction of 20.3% compared with the predicted mean in 2021. In 2022, the surgical volume returned to the predicted trend. For myringotomies (Figure 2E), the recovery during 2022 was very slight, with only 44.6% of the procedures performed compared with the prediction. This figure was lower than that in 2020 during the pandemic (a reduction of 37.6%) but greater than that in 2021 (53.7% of the prediction).

For maxillary punctures, the decline continued throughout the pandemic, with 45.2% fewer procedures than predicted in 2020, 51.6% in 2021, and 65.1% in 2022 (Figure 2E). Compared with the predicted values, the tonsillectomy (Figure 2B) and peritonsillar abscess incision (Figure 2C) volumes were reduced by 26.8% and 23.4%, respectively, in 2020. The decline continued in 2021 and 2022, with 30.0% and 37.4% fewer tonsillectomies and 28.8% and 40.8% fewer peritonsillar abscess incisions, respectively, than expected.

### 3.2. Noninfectious Procedure Volumes

**Figure 3:**
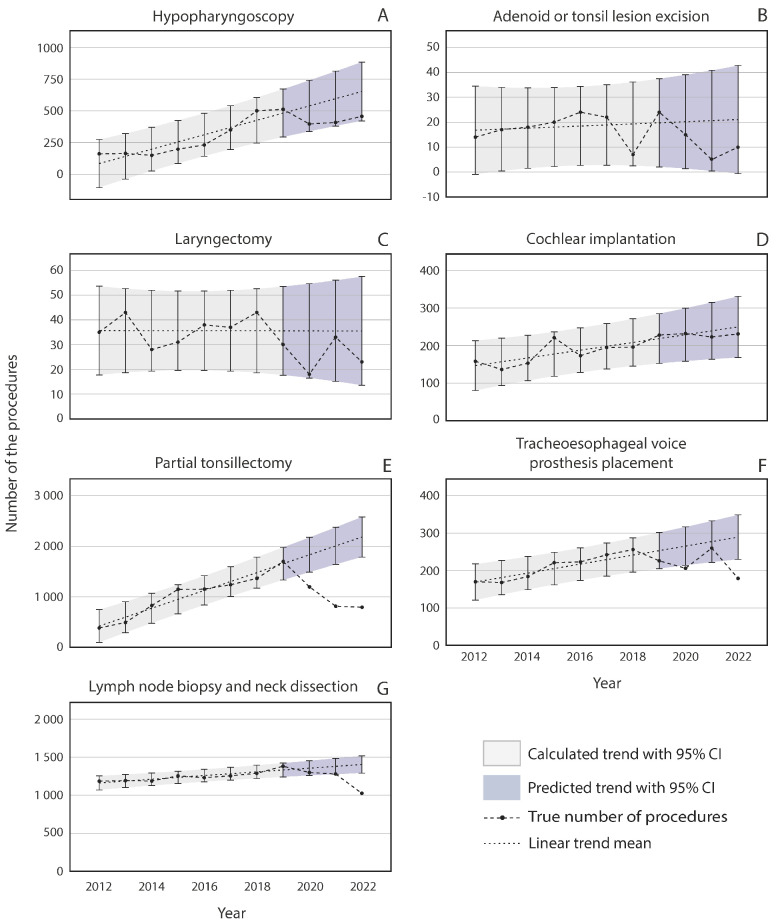
The annual number of non-infection-related otolaryngologic procedures performed in Finland from 2012 to 2022.

The annual volumes of most non-infection-related procedures, such as partial tonsillectomies, cochlear implantations, hypopharyngoscopies, cervical lymph node biopsies, and the placements of tracheoesophageal voice prostheses, slowly increased in Finland before the COVID-19 pandemic, as shown in Figure 3. The only exceptions were laryngectomies and excisions of adenoid or tonsillar lesions, which remained relatively stable.

During the pandemic years, no significant change was observed compared with the pre-pandemic trend in the number of hypopharyngoscopies, adenoid or tonsil lesion excisions, laryngectomies, or cochlear implantations (Figure 3A–D). Partial tonsillectomies and, to a lesser degree, placements of tracheoesophageal voice prostheses and cervical lymph node biopsies diverged from the predicted trend during the pandemic years (Figure 3E–G). A lesser, non-significant decrease was observed in every noninfectious procedure during the pandemic, except cochlear implantations, which stayed on an increasing trend throughout the pandemic (Figure 3). Cervical lymph node excision volumes were stable in 2020 and 2021, but in 2022, a 27% reduction was observed. The partial tonsillectomy procedure volume decreased by 34.7% in 2020, by 59.6% in 2021, and by 63.7% in 2022 (Figure 3E). Compared with the predicted trend, the prevalence of placements of tracheoesophageal voice prostheses was barely significantly lower (21.1%) during 2020 but rose back to the prediction in 2021, with a decrease in 2022 to 38.1% of the predicted trend (Figure 3F).

### 3.3. Procedures with Infectious and Noninfectious Indications

**Figure 4:**
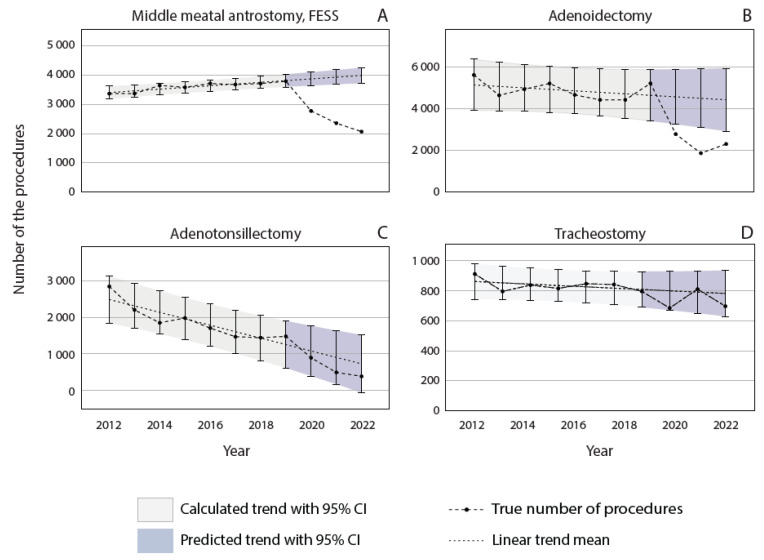
The annual number of otolaryngologic procedures performed for both infectious and noninfectious indications in Finland from 2012 to 2022.

Figure 4 illustrates the trends of uncategorized procedures in Finland from 2012 to 2022. Adenoidectomies, adenotonsillectomies, maxillary antrostomies, and tracheostomies were labeled uncategorized since their indications could not be exclusively categorized as either infection-related or nonrelated. Before the COVID-19 pandemic, the number of MMAs increased (Figure 4A), whereas adenotonsillectomies demonstrated a decreasing trend (Figure 4B). Adenoidectomies and tracheostomies were rather stable (Figure 4C,D). Compared with the predicted trend, significantly fewer endoscopic maxillary antrostomies were performed during the pandemic, with reduced volumes of 28.3% in 2020, 40.0% in 2021, and 48.2% in 2022 (Figure 4A). A similar trend was observed for adenoidectomies, with rates of 39.2%, 59.0%, and 48.0% from 2020 to 2022, respectively (Figure 4C). Adenotonsillectomies and tracheostomy procedure volumes were associated with the predicted trend during the pandemic (Figure 4B–D).

### 3.4. Comparison of Infection-Related Procedures to Non-Infection-Related Procedures

To study whether the pandemic time affected procedure volumes differently in procedures related to infections vs. non-infection-related, we calculated a residual, i.e., the difference between the observed and predicted procedure rates. The difference between the two groups indicates that the trend in surgical volumes was different between the infectious and noninfectious procedures. The number of infection-related procedures decreased significantly more than the number of non-infection-related otolaryngologic procedures in 2020 (Mann–Whitney U test, *p* = 0.026). The difference between these two categories was sustained through 2021 (*p* = 0.038). In 2022, no statistically significant difference was detected between the groups. Using the Chi-Squared test, a statistically significant difference between the two groups was observed in 2020 (*p* < 0.001), 2021 (*p* < 0.001), and 2022 (*p* = 0.002). 

## 4. Discussion

This study analyzed Finnish national health care records before (2012–2019), during (2020–2021), and after (2022) the COVID-19 pandemic to understand how the pandemic and preventive infection control measures affected long-term trends in ENT surgery volumes. The results revealed that the pandemic altered the trends of many ENT procedures, and during the pandemic, the number of infection-related surgeries declined more than non-infection-related surgeries did. The decline in infection-related surgery volumes seems to have lasted longer than the COVID-19 pandemic itself, as only tympanostomies and mastoidectomies of all the infection-related procedures included in this study have returned to the pre-pandemic trend. Some non-infection-related procedures and procedures with mixed indications also declined during the pandemic and sustained their reduction even in 2022.

This study revealed that the number of infection-related surgeries declined more than non-infection-related surgeries did. The findings imply that the decline in circulating respiratory viruses during the pandemic reduced the need for infection-related procedures. Demonstrative examples include tympanostomies and myringotomies, which are performed due to recurrent acute otitis media or chronic otitis media with effusion [22]. The annual number of tympanostomies has been decreasing steadily annually in Finland, but the pandemic has induced an extra decrease, which differs statistically significantly from the pre-pandemic trend. The long-term downward trend is probably at least partially related to the steady decrease in the number of births and thus the number of small children in Finland [23]. In addition, pneumococcal vaccination, which has been part of the national vaccination program since 2007, has decreased the incidence of otitis media (OM) [24], as *Streptococcus pneumoniae* is one of the most important pathogens of middle ear infections. Furthermore, smoking, a well-known risk factor for middle ear infections [25], has decreased throughout this time period in Finland [26]. A decline in tympanostomies was reported in the US between 2005 and 2019, in a study examining otolaryngology residents’ caseloads [27]. A similar long-term downward trend was not observed in the number of myringotomies, probably since they were performed mainly on adults. The decrease in the number of tympanostomies related to the pandemic is in line with several previous studies demonstrating similar changes [5,7,28,29]. Additionally, a growing body of evidence demonstrates the impact of social isolation on middle ear infections. In particular, quarantine periods have been associated with the resolution of OM, especially its acute and chronic forms [28,30,31]. Additionally, the volumes of all other infection-related ENT surgeries included in this study, except incisions for deep cervical abscesses, decreased during the pandemic. The number of peritonsillar abscess incisions has steadily increased over the long term. A similar long-term increase was observed in the UK over a ten-year period, which could be related to fewer tonsillectomy operations and reduced antibiotic prescribing in primary care [32]. However, they decreased steeply toward the end of the pandemic, probably due to a lower circulation of *Streoptococcus pyogenes*, as it has been identified as a key etiological factor in peritonsillar abscess formation [33]. This result is in line with data from the whole Korean population, which revealed decreases in the numbers of peritonsillar, retropharyngeal, and parapharyngeal abscess diagnoses during the first year of the pandemic compared with those before the pandemic [34]. On the other hand, a study from the US reported no difference in the number of peritonsillar abscesses from the beginning of the pandemic through the end of 2021 compared with 2019 [35]. Maxillary punctures have also been substantially affected by the pandemic. They are performed to help with diagnosis and to relieve symptoms in acute maxillary sinus infections. The evidence that maxillary punctures relieve sinusitis symptoms is not well established; hence, maxillary punctures are rarely used in other countries. The lack of evidence for their benefits could be an explanatory factor for the long-term decline. In addition to reduced infections during the pandemic leading to a further decrease in maxillary punctures, the decrease could be potentiated by reluctance to perform procedures with controversial efficacy during the pandemic. Another reason is that aerosol-producing procedures, such as maxillary punctures, were restricted during the pandemic. The rather stable long-term trend in the increase in mastoidectomies was momentarily interrupted during the pandemic.

As an exception to other infection-related procedures, the number of incisions for deep cervical abscesses tended to increase during the pandemic. This could be related to the fact that a large proportion of deep neck infections have an odontogenic etiology, and during the pandemic, access to dental care was very limited. The reason for the long-term increasing trend of deep neck infections is unclear but might be related to the aging of the population and an increase in the number of comorbidities [36]. In the future, it will be interesting to determine whether the number of deep neck infections will show a delayed increase from the trendline because of poor dental care during the pandemic.

While the idea that reduced infectious disease is the reason for the decline in surgical volume is persuasive, it does not completely explain these results. For example, in the category of non-infection-related procedures, partial tonsillectomies declined both during and after the pandemic. Tonsillectomies are performed due to infections, whereas partial tonsillectomies are mostly related to obstructive disorders [37]. Adenoidectomies and adenotonsillectomies are performed because of both indications. Considering our hypothesis of a reduced need for infection-related surgeries, it would have been expected that tonsillectomies would reduce and that partial tonsillectomies would remain rather stable. In Germany, the first lockdown resulted in an abrupt decrease in the national levels of tonsillectomies, adenotonsillectomies, and partial tonsillectomies. The levels remained lower after the lockdown, compared to 2019 [6]. A noticeable decrease in the number of adenotonsillectomies during the beginning of the COVID-19 pandemic was also detected in 51 children’s hospitals in the US. Adenotonsillectomies sustained their reduction throughout 2020 and 2021, without signs of recovery [35]. One reason for the reduction in tonsillar surgery during the pandemic might be the capacity of health care, which prioritizes more urgent procedures. Some of the reduction in adenoidectomies could be explained by a decrease in infection-caused adenoid hypertrophy [38]. Additionally, the long-term trend in the use of tonsillectomies, adenotonsillectomies, and adenoidectomies has been declining. The only exceptions are partial tonsillectomies, which have been increasing in the long term. Partial tonsillectomies are currently preferred over a tonsillectomy for the surgical management of children with sleep apnea and snoring, which could explain this trend. Additionally, the criteria for tonsillar surgery were tightened during this period.

In addition to reduced infections contributing to a decrease in surgery volume, other explanatory factors, such as health care prioritization and differences in health-seeking behavior, could be influential. More chronic conditions, such as chronic sinusitis, which is the main indication for an MMA [39], might not have been an equal concern during the pandemic. This could be one reason why the long-term upward trend in MMAs shifted into a steeply declining trend during these three years. The pandemic has increased waiting times for surgery in Finland [40]. Additionally, a reduction in the number of referrals to ENT doctors was also observed early in the pandemic [29]. This could imply altered access to primary care or changes in health-seeking behavior. This still does not reflect the number of elective surgeries, such as cochlear implantation, laryngectomies, and adenoid or tonsil lesion excision. In our study, of the noninfectious procedures, cochlear implantations were the only surgeries that were not affected by the pandemic in 2020. Especially in children, implantation could not be postponed by months since children with congenital, severe-to-profound hearing loss can only develop near-normal language skills if they are implanted early. Further, cochlear implantation surgeries have been shown to be safe and effective even during the pandemic, given that proper precautionary measures are taken [41]. In other countries, delays in these surgeries have been reported. According to one study analyzing manufacturing data, cochlear implantation in adults decreased in the US by 10.1% between 2019 and 2020. The decrease was less pronounced among children. A total of 2.2% of those aged <3 years and 3.8% of those aged 4–17 years were included [42]. Significant delays between cochlear implant evaluation and surgery were reported in a single tertiary center in the US among patients with prelingual deafness [43] as well as in lower esophageal cancer diagnosis and in endoscopic diagnostics between March 2020 and December 2020 compared to 2019 [44]. Cancer diagnoses have been delayed in Finland because head and neck cancer symptoms have been interpreted as symptoms of COVID-19 infection [45]. This type of diagnostic delay during the pandemic could have influenced cancer-related surgeries with a delay, which was reflected by the number of neck dissections and lymph node biopsies, which decreased from the trend only in 2022. The same explanation could be applied to tracheoesophageal voice prosthesis surgeries for laryngeal cancer patients, which slightly decreased from the long-term trend in 2020 and more clearly in 2022. Contrary to ENT cancer, which may have similar symptoms to respiratory infections, a study reported an increase in diagnosed basal cell carcinoma in the skin of the head and neck region and no reduction in the diagnoses of all other non-melanoma skin cancers during the pandemic. These findings were attributed to the noticeable lesions in the head and neck area that encouraged patients to seek medical care [46]. In the future, it will be interesting to see whether there will be a delayed increase in the cancer-related surgeries that have now decreased in Finland, such as neck dissections, lymph node biopsies, and tracheoesophageal voice prosthesis surgeries.

In the US, otolaryngological surgery volumes quickly rebounded in September 2020 to 99.7% of the 2019 levels of inpatient volumes and 96.5% of outpatient volumes nationwide, after the initial decrease in the early pandemic [47]. However, significant differences among the subspeciality surgery volumes have been reported. A study conducted in the US found out that two years from the onset of the pandemic, the only subspecialities that did not exhibit a rebound in surgery volumes were pediatric otolaryngology and otology [48]. Others such as general surgery, endoscopy, rhinology, laryngology, facial plastics and reconstructive surgery, and head and neck surgery have returned to the pre-pandemic levels. This is in line with our findings that common pediatric otolaryngological procedures such as tympanostomies, adenoidectomies, and tonsillectomies have not returned to the pre-pandemic trend. The researchers reported non-significant increases only in emergency endotracheal intubations and incision procedures of the trachea during the post-pandemic period among otolaryngological procedures [48]. However, the volumes of some procedures have even surpassed the pre-pandemic trend. A register study in the US reported an increase in laryngological procedures and a shift to office-based procedures after the onset of the pandemic that was sustained throughout 2022 [49]. Also, the volumes of head and neck microvascular free flap surgery have increased and surpassed the pre-pandemic volumes [50].

In our study, surgery volumes did not fully recover even after the pandemic time was over. In June 2023, COVID-19 was removed from the list of generally hazardous communicable diseases in Finland [51], but respiratory viruses returned to the circulation earlier. The influenza season from 2021 to 2022 began normally, although the epidemic peak was delayed to May 2022 due to restrictions imposed by the Omicron variant [52]. RSV experienced a substantial epidemic in the winter between 2020 and 2021, and a second epidemic began around October 2022 [53]. There are multiple possible reasons for the continuing decline in surgical volumes. A delay in the resumption of infection-related surgical volumes would be expected, since the indications require a certain number of consecutive infections, which the pandemic interrupted. However, this does not explain why tympanostomies and mastoidectomies are the only infection-related procedures that have returned to pre-pandemic trends. Age could be another explanatory factor, since the highest incidence of OM is among young children of the age of 6–24 months [54], whose social restrictive measures were loose compared with those of older children and adults. Primary care centers were closed in Finland only during the first wave of the pandemic (Figure 1). In addition, compliance with NPIs is presumably higher for kindergarten-aged children. Another age-related explanatory factor could be related to the time delay between diagnosis and operative management. As children typically undergo surgery more swiftly, it is intuitive that tympanostomies have recovered more quickly since the pandemic. Third, the pandemic might have had a longer lasting effect on surgery indications, i.e., a more conservative approach in some ENT diseases, such as punctures of the maxillary sinuses, although it is worth mentioning that the management guidelines have remained unchanged. This could also explain the prolonged decline in some of the non-infection-related surgery volumes, such as those of partial tonsillectomies and surgeries with mixed indications, such as MMAs and adenoidectomies.

We acknowledge some limitations of the study. In the statistical analysis, we assume a linear trend for the number of procedures but can, of course, not be certain that it would have been linear without the COVID-19 pandemic. Another potential limitation is the accuracy of the reporting of the procedures, even though health care providers in Finland are legally obliged to report procedures.

## 5. Conclusions

In conclusion, this study provides a nationwide insight into ENT surgery volumes in Finland over a ten-year period and shows that the COVID-19 pandemic caused a significant decline in ENT surgical volumes, with varying rates of recovery across subspecialties. While some areas have recovered to or exceeded pre-pandemic levels, others continue to experience reduced volumes, highlighting the lasting impact of the pandemic on ENT surgical practice. Although causative reasoning cannot be conducted based on this study, it still provides a good indication of how the absence of respiratory viruses and multifactorial societal restriction measures could have a long-lasting effect on the epidemiology and management of many ENT diseases.

## Figures and Tables

**Table 1:** Volumes of otolaryngologic procedures performed in Finland from 2012 to 2022. The procedures are classified by the Nordic Classification of Surgical Procedures (NCSP) codes. The surgeries were categorized into those related to infections, those not related, and those that were performed in both situations and thus excluded from the comparisons.

NCSP Code	Procedure	Infection-Related	2012	2013	2014	2015	2016	2017	2018	2019	2020	2021	2022	Total
UJC01	Hypopharyngoscopy	No	161	164	149	198	231	350	501	513	397	409	457	3530
EMB00	Excision of lesion of tonsil or adenoids	No	14	17	18	20	24	22	7	24	15	5	10	176
PJD41, PJD51, PJD91, PJD71	Cervical lymph node biopsy and neck dissection	No	1185	1191	1186	1253	1230	1254	1288	1381	1298	1279	1024	13,569
DQB20, DQB30, DQB40, DQB50	Partial or total laryngectomy	No	35	43	28	31	38	37	43	30	18	33	23	359
DQE00	Placement of tracheoesophageal voice prosthesis	No	170	168	184	221	223	242	256	226	206	260	179	2335
DFE00	Cochlear implantation	No	158	136	153	221	173	195	196	228	232	223	231	2146
EMB15	Partial tonsillectomy	No	382	490	825	1147	1151	1238	1364	1697	1195	811	792	11,092
EMB10	Tonsillectomy	Yes	6283	5955	5495	5686	5504	5090	5348	5538	3730	3465	3047	55,141
DCA20	Tympanostomy	Yes	14,872	12,356	12,419	13,161	11,272	9512	9041	9838	4559	2420	3963	103,413
DEB00, DEB10, DEB20, DEB25	Mastoidectomy	Yes	207	215	223	199	228	204	232	216	148	178	181	2231
TDM10	Puncture or lavage of maxillary sinus	Yes	6859	6175	5803	6591	6452	6212	6152	5936	3278	2860	2039	58,357
DCA10	Myringotomy	Yes	1659	1536	1681	1860	1866	1643	1780	1768	1138	856	1036	16,823
ENA32	Incision of deep cervical abscess	Yes	160	186	164	143	180	213	237	270	223	249	328	2353
ENA00	Incision of peritonsillar abscess	Yes	241	319	420	613	655	647	706	789	685	692	620	6387
EMB30	Adenoidectomy	Excluded	5612	4636	4929	5203	4657	4416	4418	5210	2777	1860	2300	46,018
DMB20	Middle meatal antrostomy	Excluded	3368	3373	3650	3585	3717	3682	3717	3794	2776	2357	2067	36,083
EMB20	Adenotonsillectomy	Excluded	2850	2206	1852	1984	1708	1471	1444	1482	894	494	385	16,770
GBB00	Tracheostomy	Excluded	-	912	796	838	816	847	842	796	685	811	697	8040

## Data Availability

The data are freely available from the Finnish Institute for Health and Welfare (THL), https://sampo.thl.fi/pivot/prod/fi/thil/perus01/fact_thil_perus01 (accessed on 22 March 2023).

## Appendix A

On 16 March 2020, a state of emergency was declared, leading to a national lockdown. Public premises, schools, and universities were closed, and gatherings of more than 10 people were prohibited. Finland’s borders were closed from noncitizens, and traveling abroad was prohibited. Social distancing, hand washing, and proper sneezing techniques were recommended. The use of a face mask in public places was not encouraged at that time [16]. Two months later, on the 14 May, Finland moved to a “hybrid strategy”, which meant milder restrictions, effective testing, and the isolation of those exposed to COVID-19. A gathering of 50 people was allowed. In July, the limit was increased to 500, and at the end of August 2020, limitations were lifted, travel restrictions were loosened, and schools and daycares were reopened [17]. Instead, using face masks if social distancing was impossible was recommended for those over 15 years of age [18]. With the increase in hospitalizations, restrictions were implemented more regionally starting on 16 September 2020 and were based on three categories of COVID-19 epidemiological activity (the base level, acceleration stage, and spreading stage) [19].

In December 2020, the first COVID-19 vaccines were dispensed in Finland [20]. By November 2021, the coverage was over 80% for the first vaccine. In February 2021, a state of emergency was again declared. Districts that were in the acceleration stage went under a lockdown similar to that during 2020 [21]. Regional restrictions of varying severity were continued until February 2022, after which restriction measures were lifted.

## References

[1] Saraswathula A., Shippey E., Sprankle L.A., Kachalia A., Miller R.G., Gourin C.G., Stewart C.M. (2022). Comparison of subspecialty major surgical volume in the United States during the COVID-19 pandemic. Laryngoscope.

[2] Hervochon R., Atallah S., Levivien S., Teissier N., Baujat B., Tankere F. (2020). Impact of the COVID-19 epidemic on ENT surgical volume. Eur. Ann. Otorhinolaryngol. Head Neck Dis..

[3] Mattingly A.S., Rose L., Eddington H.S., Trickey A.W., Cullen M.R., Morris A.M., Wren S.M. (2021). Trends in US Surgical Procedures and Health Care System Response to Policies Curtailing Elective Surgical Operations during the COVID-19 Pandemic. JAMA Netw. Open.

[4] Kourelis K., Angelopoulou M., Goulioumis A., Fouzas S., Kourelis T. (2022). Surgery for adenotonsillar hypertrophy and otitis media in children is less demanded in quarantine times. Int. J. Pediatr. Otorhinolaryngol..

[5] Allen D.Z., Challapalli S., McKee S., Lee K.H., Bell C.S., Roy S., Bowe S., Balakrishnan K., Chang C.W.D., Huang Z. (2022). Impact of COVID-19 on nationwide pediatric otolaryngology: Otitis media and myringotomy tube trends. Am. J. Otolaryngol..

[6] Windfuhr J.P., Günster C. (2022). Impact of the COVID-pandemic on the incidence of tonsil surgery and sore throat in Germany. Eur. Arch. Oto-Rhino-Laryngol..

[7] Diercks G.R., Cohen M.S. (2022). The Effect of the COVID-19 Pandemic on Pediatric Tympanostomy Tube Placement. Otolaryngol. Neck Surg..

[8] Olsen S.J., Winn A.K., Budd A.P., Prill M.M., Steel J., Midgley C.M., Kniss K., Burns E., Rowe T., Foust A. (2021). Changes in influenza and other respiratory virus activity during the COVID-19 pandemic—United States, 2020–2021. Am. J. Transplant..

[9] Rankin D.A., Spieker A.J., Perez A., Stahl A.L., Rahman H.K., Stewart L.S., Schuster J.E., Lively J.Y., Haddadin Z., Probst V. (2023). Circulation of Rhinoviruses and/or Enteroviruses in Pediatric Patients with Acute Respiratory Illness Before and during the COVID-19 Pandemic in the US. JAMA Netw. Open.

[10] Groves H.E., Piché-Renaud P.-P., Peci A., Farrar D.S., Buckrell S., Bancej C., Sevenhuysen C., Campigotto A., Gubbay J.B., Morris S.K. (2021). The impact of the COVID-19 pandemic on influenza, respiratory syncytial virus, and other seasonal respiratory virus circulation in Canada: A population-based study. Lancet Reg. Health–Am..

[11] Kuitunen I., Artama M., Mäkelä L., Backman K., Heiskanen-Kosma T., Renko M. (2020). Effect of Social Distancing Due to the COVID-19 Pandemic on the Incidence of Viral Respiratory Tract Infections in Children in Finland during Early 2020. Pediatr. Infect. Dis. J..

[12] Kelloniemi S., Heikkilä P., Palmu S. (2021). COVID-19 restrictions probably brought the 2019–2020 Finnish influenza season to an early end and led to fewer respiratory viruses among infants. Acta Paediatr..

[13] Kuitunen I., Artama M., Haapanen M., Renko M. (2022). Respiratory virus circulation in children after relaxation of COVID-19 restrictions in fall 2021—A nationwide register study in Finland. J. Med. Virol..

[14] Kuitunen I., Artama M., Haapanen M., Renko M. (2021). Rhinovirus spread in children during the COVID-19 pandemic despite social restrictions—A nationwide register study in Finland. J. Med. Virol..

[15] Le Bihan-Benjamin C., Rocchi M., Putton M., Méric J.-B., Bousquet P.J. (2023). Estimation of Oncologic Surgery Case Volume Before and After the COVID-19 Pandemic in France. JAMA Netw. Open.

[16] Government Communications Department, Ministry of Education and Culture, Ministry of Social Affairs and Health. https://valtioneuvosto.fi/en/-/10616/hallitus-totesi-suomen-olevan-poikkeusoloissa-koronavirustilanteen-vuoksi?languageId=en_US.

[17] Finnish Government. https://valtioneuvosto.fi/paatokset/paatos?decisionId=0900908f806a9de2.

[18] Finnish Institute for Health and Welfare. https://thl.fi/fi/-/thl-suosittaa-kasvomaskin-kayttoa-toisten-suojaamiseksi-kasienpesu-ja-turvavalit-ovat-tarkeimmat-keinot-ehkaista-koronatartuntoja.

[19] Government Communications Department Ministry of Social Affairs and Health. https://valtioneuvosto.fi/en/-//10616/government-discusses-action-plan-to-manage-covid-19.

[20] Hartonen T., Jermy B., Sõnajalg H., Vartiainen P., Krebs K., Vabalas A., Metspalu A., Esko T., Nelis M., Hudjashov G. (2023). Nationwide health, socio-economic and genetic predictors of COVID-19 vaccination status in Finland. Nat. Hum. Behav..

[21] Government Communications Department. https://valtioneuvosto.fi/en/-//10616/government-decides-on-immediate-transition-to-tier-2-of-covid-19-prevention-and-is-prepared-to-declare-a-state-of-emergency.

[22] Rosenfeld R.M., Schwartz S.R., Pynnonen M.A., Tunkel D.E., Hussey H.M., Fichera J.S., Grimes A.M., Hackell J.M., Harrison M.F., Haskell H. (2013). Clinical Practice Guideline: Tympanostomy Tubes in Children. Otolaryngol. Neck Surg..

[23] Vastasyntyneet Alueittain—THL Kuutio-ja Tiivistekäyttöliittymä. https://sampo.thl.fi/pivot/prod/fi/synre/vastasyalue/fact_synre_vastasyalue.

[24] Prymula R., Peeters P., Chrobok V., Kriz P., Novakova E., Kaliskova E., Kohl I., Lommel P., Poolman J., Prieels J.-P. (2006). Pneumococcal capsular polysaccharides conjugated to protein D for prevention of acute otitis media caused by both Streptococcus pneumoniae and non-typable Haemophilus influenzae: A randomised double-blind efficacy study. Lancet.

[25] DiFranza J.R., Lew R.A. (1996). Morbidity and Mortality in Children Associated with the Use of Tobacco Products by Other People. Pediatrics.

[26] Finnish Institute for Health and Welfare Tobacco Statistics 2022. https://thl.fi/en/statistics-and-data/statistics-by-topic/alcohol-drugs-and-addiction/tobaccostatistics.

[27] Welschmeyer A., Coerdt K., Crossley J., Malekzadeh S. (2021). Critical Evaluation of Trends in Otolaryngology Resident Caseload by Subspecialty from 2005 to 2019. Ann. Otol. Rhinol. Laryngol..

[28] Pereira N.M., Maresh A.M., Modi V.K., Rosenblatt S.D. (2022). Tympanostomy tubes in the age of quarantine. Int. J. Pediatr. Otorhinolaryngol..

[29] Haapanen M., Renko M., Artama M., Manninen I., Mattila V.M., Uimonen M., Ponkilainen V., Kuitunen I. (2021). Tympanostomies and tonsillar surgery in children during the COVID-19 pandemic in Finland. Laryngoscope Investig. Otolaryngol..

[30] Aldè M., Di Berardino F., Marchisio P., Cantarella G., Ambrosetti U., Consonni D., Zanetti D. (2021). Effects of COVID-19 Lockdown on Otitis Media with Effusion in Children: Future Therapeutic Implications. Otolaryngol. Neck Surg..

[31] Torretta S., Capaccio P., Coro I., Bosis S., Pace M.E., Bosi P., Pignataro L., Marchisio P. (2021). Incidental lowering of otitis-media complaints in otitis-prone children during COVID-19 pandemic: Not all evil comes to hurt. Eur. J. Pediatr..

[32] Powell J., Wilson J.A. (2012). An evidence-based review of peritonsillar abscess. Clin. Otolaryngol..

[33] Saar M., Vaikjärv R., Parm Ü., Kasenõmm P., Kõljalg S., Sepp E., Jaagura M., Salumets A., Štšepetova J., Mändar R. (2023). Unveiling the etiology of peritonsillar abscess using next generation sequencing. Ann. Clin. Microbiol. Antimicrob..

[34] Kim S.Y., Yoo D.M., Kim J.H., Kwon M.J., Kim J.H., Chung J., Choi H.G. (2022). Changes in Otorhinolaryngologic Disease Incidences before and during the COVID-19 Pandemic in Korea. Int. J. Environ. Res. Public Health.

[35] Allen D.Z., Challapalli S., Lee K.H., Bell C.S., Roy S., Bowe S., Balakrishnan K., Chang C.W.D., Huang Z. (2022). Impact of COVID-19 on nationwide pediatric otolaryngology practice: Adenotonsillectomies (TA) and tonsil-related diagnoses trends. Am. J. Otolaryngol..

[36] Velhonoja J., Lääveri M., Soukka T., Irjala H., Kinnunen I. (2020). Deep neck space infections: An upward trend and changing characteristics. Eur. Arch. Oto-Rhino-Laryngol..

[37] Blackshaw H., Springford L.R., Zhang L.-Y., Wang B., Venekamp R.P., Schilder A.G.M. (2020). Tonsillectomy versus tonsillotomy for obstructive sleep-disordered breathing in children. Cochrane Database Syst. Rev..

[38] Niedzielski A., Chmielik L.P., Mielnik-Niedzielska G., Kasprzyk A., Bogusławska J. (2023). Adenoid hypertrophy in children: A narrative review of pathogenesis and clinical relevance. BMJ Paediatr. Open.

[39] Fokkens W.J., Lund V.J., Hopkins C., Hellings P.W., Kern R., Reitsma S., Toppila-Salmi S., Bernal-Sprekelsen M., Mullol J., Alobid I. (2020). European Position Paper on Rhinosinusitis and Nasal Polyps 2020. Rhinol. J..

[40] Uimonen M., Kuitunen I., Ponkilainen V., Mattila V.M. (2022). Prioritizing Elective Surgery during the COVID-19 Pandemic Has Caused Age-Related Inequality: A Multicenter Study. SN Compr. Clin. Med..

[41] Skarzynski H., Lorens A., Dziendziel B., Wlodarczyk E., Obrycka A., Walkowiak A., Skarzynski P.H. (2021). Resumption of Cochlear Implant Surgery under COVID-19 Pandemic Conditions. Life.

[42] Marinelli J.P., Nassiri A.M., Lohse C.M., Driscoll C.L.W., Neff B.A., Carlson M.L. (2023). Effect of a Global Pandemic on Adult and Pediatric Cochlear Implantation across the United States. Otol. Neurotol..

[43] Noij K.S., Huang E.Y., Walsh J., Creighton F.X., Galaiya D., Bowditch S.P., Stewart C.M., Jenks C.M. (2023). Trends in Timing and Provision of Pediatric Cochlear Implant Care during COVID-19. OTO Open.

[44] Khan H., Johnson C., Malwankar J., Battafarano R., Yang S., Broderick S., Huang P., Lam V., Ha J. (2023). The COVID-19 Era Is Associated with Delays in Esophageal Cancer Diagnosis and Treatment. J. Surg. Res..

[45] Paajanen J., Mäkinen L.K., Suikkila A., Rehell M., Javanainen M., Lindahl A., Kekäläinen E., Kurkela S., Halmesmäki K., Anttila V.-J. (2021). Isolation precautions cause minor delays in diagnostics and treatment of non-COVID patients. Infect. Prev. Pract..

[46] Benedetti S., Frosolini A., Catarzi L., Marsiglio A., Gennaro P., Gabriele G. (2024). Impact of the COVID-19 Pandemic on the Diagnosis and Management of Non-Melanoma Skin Cancer in the Head and Neck Region: A Retrospective Cohort Study. Healthcare.

[47] Saraswathula A., Gourin C.G., Stewart C.M. (2021). National Trends in US Otolaryngology Surgical Volume during the Early COVID-19 Pandemic. JAMA Otolaryngol. Head Neck Surg..

[48] Qatanani A.M., Kshirsagar R.S., Douglas N.O., Andrade C., Adappa N.D., Eide J.G. (2022). Trends in otolaryngology-head and neck surgery procedural volumes during the COVID-19 pandemic. Int. J. Otorhinolaryngol. Head Neck Surg..

[49] Gray R., Ryan M.A., Mehta V. (2024). Volume and Practice-Setting Shift of Laryngology Procedures during the COVID-19 Pandemic: A Reg-ENT Database Analysis. OTO Open.

[50] Wu S., Shirley R.B., Genther D., Ciolek P., Prendes B., Hopkins B., Ku J. (2023). Trends in Surgical Volume and Postoperative Outcomes of Head and Neck Free Flap Reconstruction Using the Finance-Electronic Medical Record Digital Dashboard. J. Clin. Otorhinolaryngol..

[51] Ministry of Social Affairs and Health. https://valtioneuvosto.fi/en/-//1271139/covid-19-no-longer-classified-as-generally-hazardous-communicable-disease.

[52] Kuitunen I., Renko M., Tapiainen T. (2022). Unusual late epidemic peak during influenza season 2021–2022: A nationwide register-based analysis in Finland. Influenza Other Respi. Viruses.

[53] Finnish Institute for Health and Welfare. https://thl.fi/fi/web/infektiotaudit-ja-rokotukset/taudit-ja-torjunta/taudit-ja-taudinaiheuttajat-a-o/rsv/rsv-esiintyvyys-suomessa.

[54] Acute Otitis Media (Children) Current Care Guidelines. Working Group Set Up by the Finnish Medical Society Duodecim, Finnish Association of Otorhinolaryngology—Head and Neck Surgery, Finnish Paediatric Society, Finnish General Medicine Society. Helsinki. https://www.kaypahoito.fi/hoi31050#K1.

